# Challenges in Implementing CAUTI Surveillance in Resource-Constrained Settings: Lessons from a Kenyan Referral Hospital

**DOI:** 10.1017/ash.2025.198

**Published:** 2025-09-24

**Authors:** Flavia Nakayima Miiro, Kate Ellingson, Linus Ndegwa, Irene Muramba

**Affiliations:** 1 University of Arizona; 2 University of Arizona, College of Public Health; 3 Centres for Disease Control- CDC; 4 Coast General Teaching and Referral Hospital

## Abstract

**Background:** Catheter-associated urinary tract infections (CAUTIs) are a challenge for hospitalized patients accounting for approximately 40% of all healthcare-associated infections. CAUTI surveillance remains underdeveloped in many Sub-Saharan countries, even though identifying infections is critical to prevention and management. Standardized CAUTI surveillance among 45 LMICs conducted in intensive care units (ICUs) has demonstrated high CAUTI incidence compared to high-income countries. However, few studies have examined CAUTI in non-ICU settings in LMIC, where catheter use is common. We aimed to identify challenges in CAUTI surveillance related to documentation and antibiotic use patterns among adult inpatients in non-ICU wards in a Kenyan public hospital. **Methods:** Using a cross-sectional design, we retrospectively abstracted data on non-ICU adult inpatients from clinical and laboratory records. We identified patients with suspected UTI through urine culture requests from 1/1/2023-12/31/2023, whom we linked to clinical records. We abstracted data on diagnosis on admission, socio-demographics, urinary catheter indication and duration, UTI symptoms, urine culture results, and antibiotic use. This descriptive analysis summarizes characteristics of patients with suspected UTI to identify factors hindering CAUTI surveillance in non-ICU settings. **Results:** 293 non-ICU adult inpatients admitted to Mombasa Regional Referral Hospital had at least one urine culture request in 2023. Of these 193 (65.9%) had indwelling urinary catheters (IUC) inserted. Among those with IUC, 49.7% were female, with an average age of 51.5 years, with majority (64.8%) admitted to the medical wards; 5.2% had no recorded indication for catheterization and 82.9% had no UTI symptoms documented in the 2 days before the urine culture request. There were 124 negative cultures, 4 were determined to be contaminated, 6 did not have results on file, and 59 were positive; pathogens identified in the positive cultures included Escherichia Coli (51.8%), Klebsiella Pneumoniae (28.6%), Pseudomonas aeruginosa (10.7%), and others (8.9%) including Klebsiella Oxytoca, Acinetobacter baumanii, and Protein Mirabilis. 38.3% were prescribed intravenous antibiotics in the 7 days before the urine culture was obtained. 66.3% had no documentation of IUC removal, and 10.9% had incomplete documentation on file with missing pages. **Conclusion:** Myriad challenges to accurate CAUTI surveillance were identified among non-ICU patients at a Kenyan regional referral hospital. Lack of documentation of clinical symptoms makes application of standard case definitions challenging, and non-documentation of catheter removal dates hinders calculation of incidence using a catheter-day denominator. Further, the administration of antibiotics prior to urine culture hinders identification of potential source pathogens. Documentation and antibiotic administration practices are major hurdles for CAUTI surveillance.

